# Next-Generation Sequencing for Confronting Virus Pandemics

**DOI:** 10.3390/v14030600

**Published:** 2022-03-14

**Authors:** Josep Quer, Sergi Colomer-Castell, Carolina Campos, Cristina Andrés, Maria Piñana, Maria Francesca Cortese, Alejandra González-Sánchez, Damir Garcia-Cehic, Marta Ibáñez, Tomàs Pumarola, Francisco Rodríguez-Frías, Andrés Antón, David Tabernero

**Affiliations:** 1 Liver Diseases-Viral Hepatitis, Liver Unit, Vall d’Hebron Institut of Research (VHIR), Vall d’Hebron Hospital Universitari, Vall d’Hebron Barcelona Hospital Campus, Passeig Vall d’Hebron 119-129, 08035 Barcelona, Spain; sergi.colomer@vhir.org (S.C.-C.); carolina.campos@vhir.org (C.C.); damir.garcia@vhir.org (D.G.-C.); marta.ibanez@vhir.org (M.I.); 2 Centro de Investigación Biomédica en Red de Enfermedades Hepáticas y Digestivas (CIBERehd), Instituto de Salud Carlos III, Av. Monforte de Lemos 3-5, 28029 Madrid, Spain; maria.cortese@vhir.org (M.F.C.); frarodri@vhebron.net (F.R.-F.); david.tabernero@vhir.org (D.T.); 3 Biochemistry and Molecular Biology Department, Universitat Autònoma de Barcelona (UAB), UAB Campus, Plaça Cívica, 08193 Bellaterra, Spain; 4 Microbiology Department, Vall d’Hebron Institut of Research (VHIR), Vall d’Hebron Hospital Universitari, Vall d’Hebron Barcelona Hospital Campus, Passeig Vall d’Hebron 119-129, 08035 Barcelona, Spain; cristina.andres@vhir.org (C.A.); maria.pinana@vhir.org (M.P.); alejandra.gonzalez@vhebron.net (A.G.-S.); tpumarola@vhebron.net (T.P.); 5 Clinical Biochemistry Research Group, Biochemistry Department, Vall d’Hebron Institut of Research (VHIR), Vall d’Hebron Hospital Universitari, Vall d’Hebron Barcelona Hospital Campus, Passeig Vall d’Hebron 119-129, 08035 Barcelona, Spain; 6 Microbiology Department, Universitat Autònoma de Barcelona (UAB), UAB Campus, Plaça Cívica, 08193 Bellaterra, Spain; 7 Microbiology Departments, Hospital Universitari Vall d’Hebron, Vall d’Hebron Barcelona Hospital Campus, Passeig Vall d’Hebron 119-129, 08035 Barcelona, Spain

**Keywords:** NGS, deep-sequencing, viruses, SARS-CoV-2, COVID-19, variability, zoonosis, pandemics, diagnostic tools

## Abstract

Virus pandemics have happened, are happening and will happen again. In recent decades, the rate of zoonotic viral spillover into humans has accelerated, mirroring the expansion of our global footprint and travel network, including the expansion of viral vectors and the destruction of natural spaces, bringing humans closer to wild animals. Once viral cross-species transmission to humans occurs, transmission cannot be stopped by cement walls but by developing barriers based on knowledge that can prevent or reduce the effects of any pandemic. Controlling a local transmission affecting few individuals is more efficient that confronting a community outbreak in which infections cannot be traced. Genetic detection, identification, and characterization of infectious agents using next-generation sequencing (NGS) has been proven to be a powerful tool allowing for the development of fast PCR-based molecular assays, the rapid development of vaccines based on mRNA and DNA, the identification of outbreaks, transmission dynamics and spill-over events, the detection of new variants and treatment of vaccine resistance mutations, the development of direct-acting antiviral drugs, the discovery of relevant minority variants to improve knowledge of the viral life cycle, strengths and weaknesses, the potential for becoming dominant to take appropriate preventive measures, and the discovery of new routes of viral transmission.

## 1. Introduction

The recent pandemic caused by Severe Acute Respiratory Syndrome Coronavirus-2 (SARS-CoV-2) has exposed the weakness of our surveillance system with regard to preventing and monitoring the emergence and spread of emerging, re-emerging or new viruses. One of the lessons that has been drawn from this pandemic is that the use of high-throughput technological tools for the early detection and rapid identification and characterization of the causal agent, is essential for controlling the worst effects of any pandemic. Currently, next-generation sequencing (NGS) technologies for metagenomics, whole-genome sequencing (WGS) and targeted deep-sequencing are the best tools for genetic identification and characterization of viral agents, for studying their variability, allowing for the correct classification of the agent, identifying viral genetic markers associated with virulence, and considering antigenicity and susceptibility to antivirals based on pre-existing knowledge where available [1,2,3,4,5]. Other ways to describe NGS include deep-sequencing, massive parallel sequencing, high-throughput sequencing, all equally valid, to differentiate it from Sanger sequencing. During the current SARS-CoV-2 pandemic, NGS technologies have allowed for the development in record time (months) of highly effective vaccines based on mRNA/DNA [6], the development of qualitative and quantitative diagnostic solutions [7], and the identification of variants and relevant mutations in continuous surveillance [8], especially of variants with evidence of mutations impacting transmissibility, severity and/or immunity, known as variants of concern (VOC), and of their dominance and spread throughout the world [9]. Early detection of VOC is important because any delayed or null identification of circulating viral agents calls into question the efficacy of a health system in preventing and responding efficiently to viral infections [10]. It is worth highlighting that it is not yet possible to identify the viral causative agent of between 60% and 80% of meningitis/encephalitis, 50% of acute gastroenteritis, 20% of haemorrhagic fever, and between 15% and 25% of acute respiratory infections in patients admitted to our hospitals [11,12,13,14,15].

Furthermore, infectious disease emergencies caused by viruses including Zika, chikungunya, human immunodeficiency virus (HIV), Crimean-Congo virus, Ebola and Marburg viruses, Influenza viruses, enteric viruses, yellow fever virus, measles virus, papillomavirus, herpesvirus, smallpox virus, polioviruses, and coronaviruses (CoV) such as SARS-CoV, Middle East respiratory syndrome coronavirus (MERS-CoV), and SARS-CoV-2, and also hepatitis C (HCV), B (HBV), E, and A viruses are becoming much more frequent than in the past. For instance, the time elapsed between the three outbreaks of CoV has been reduced, from 10 years between the first SARS-CoV outbreak (November 2002) and MERS (2012) to only 7 years until SARS-CoV-2 (December 2019) [16]. There may be multiple explanations for this reduced gap, as will be discussed later (see Section 3).

Genetic detection, identification, and characterization of infectious agents are the first steps to confront the continuous challenge they pose, as the information obtained makes it possible to develop the first molecular assays for detection of the first cases as well as to acquire valuable knowledge for the rapid development of vaccines for prevention or in silico tests of candidate direct-acting antiviral drugs for treatment. A common trait among most of these viral agents is their strong ability to generate genetic variability through different mechanisms, as will be discussed later (see Section 4). This genetic variability can affect some phenotyping features such as tropism, antigenicity, and susceptibility to available antiviral drugs. Therefore, any source of variation provides a minor viral reservoir of genomes that may be selected in the event of environmental selective pressure, such as the host immune response and the use of vaccines or antiviral drugs, thus leading viral populations to evolve [4,5,16].

We should be aware that most human viruses have a zoonotic origin (see Section 3). Such viral agents can persist and continue evolving by random mutations in their animal reservoirs, with the potential to infect humans with new variants [17]. Viruses cannot be walled-off and we can thus only tackle the threat with specific tools for the early and rapid identification of these agents, especially at the human-animal interface. An analogous strategy is applied to avoid the direct consequences of a bioterrorist movement. It is preferable to reveal the incipient evidence when only a few individuals have been recruited amongst millions of non-terrorist individuals. In the case of viruses, this surveillance task can be carried out by means of NGS technology, as has been done with hepatitis viruses [4,18].

This study reviews how NGS may be useful for dealing with viral pandemics, taking as an example the results of the application of those technologies to the study of SARS-CoV-2 quasispecies during the current coronavirus disease 2019 (COVID-19) pandemic.

## 2. NGS for Confronting the Devastating Effects of a Pandemic

Many questions arise recurrently as a pandemic progresses, namely, how is the virus evolving and genetically changing over time? Which mutations are associated with attenuation or an increase in disease severity? There is also great interest in identifying the source of new variants, whether they have appeared in a given population or have been imported. Furthermore, a diagnosis of true reinfection with SARS-CoV-2 is only reported when viral clearance is complete for the first viral infection episode [19], however, to differentiate from reactivation of the previous infection it is crucial to use NGS to prove it. Another NGS application for the recently instated vaccination campaign for COVID-19 is to use high-throughput sequencing technologies for surveillance of vaccine escape mutations to adjust development of new vaccine formulations. Another key question is related to the capacity of the viral agent to generate resistance to treatments based on direct-acting antivirals, monoclonal antibodies, or convalescent plasma. All these questions have serious social and economic consequences, representing important threats to the national health care system.

**Figure 1:**
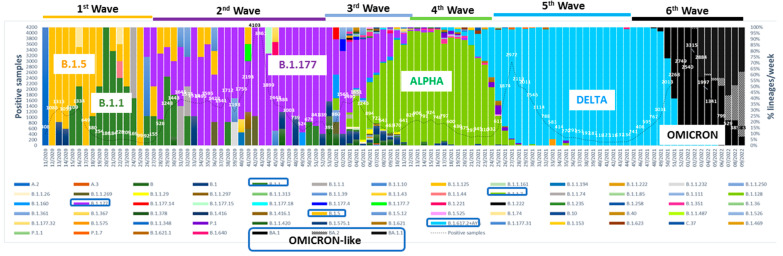
Week-by-week distribution of variants from March 2020 to present (February 2020). We have encircled the most prevalent variants in each pandemic wave. Pointed line with numbers (· · ·) indicate the amount of positive cases detected in Vall d’Hebron Hospital per week. Pointed and slashed line (· - · -) indicates vaccine coverage in Barcelona city with one dose and slashed line (- -) with double doses.

Viral genomic information is crucial to developing vaccines and antivirals and to readapting these therapies when escape or resistance mutations are identified. For instance, studying genetic changes in influenza viruses, which are constantly changing, helps to select candidate vaccine strains [20], to detect and monitor resistant variants to antiviral drugs among currently-circulating influenza viruses or strains detected in hospitalized patients under treatment [21], to identify viral factors related to virulence, as well as prevent the potential of influenza viruses from animals infecting humans. Another example of success is the polio vaccine, for which the first prototype was developed by Hilary Koprowski in 1950 but not approved [22], followed by the vaccine developed by Jonas Salk in 1952 using attenuated virus, and, finally, the attenuated Sabin vaccine given orally, composed of the three serotypes or strains, Sabin strain type 1, type 2 and type 3 [23], which has been used to nearly eradicate polio infection worldwide [24]. Poliovirus sequencing is currently performed to identify the virus, detect genetic features such as recombinant events in addition to genetic drift, propose the likely origin, track geographic patterns of spread, and determine the appropriate vaccination response [25]. The sequencing of West African Ebola outbreaks showed that all strains were related and that the virus could spread sexually [26]. Zika sequencing was able to confirm the origin and spread of the virus in the Americas with a Brazilian origin of the virus that spread through the Americas [27] and that the Caribbean was the main source of Zika in Miami, particularly from cruise ships [28]; and sequencing also revealed that the causal agent of severe respiratory illness and acute flaccid myelitis in some children in Spain in 2016 was enterovirus D68 [29]. In the case of SARS-CoV-2, it is clear that sequencing is required for continuous surveillance, for studying nosocomial or community outbreaks such as those in nursing homes or among health care workers, for confirming reinfection after a long period of negative PCR detection, identifying new variants with different transmission capabilities such as vaccine escape mutants, drug resistance genomes or viral dynamics in a chronically (long-COVID) infected patient [8,30,31,32,33,34]. For example, NGS application for viral surveillance was able to describe, at the beginning of the first wave of SARS-CoV-2 infection (Figure 1), the predominance of the variant B.1.5 (51%) in our geographic area of Barcelona until May 2020, when variant B.1.1 (33%) significantly increased, coinciding with the start of de-escalation. However, the end of the lockdown (June–July 2020) and opening of summer activities coincided with the detection of variant B.1.177, which became the predominant lineage during the entire second wave until the end of 2020. At the beginning of 2021, B.1.1.7 (Alpha variant) gradually replaced B.1.177 and became dominant during the late third (55.44%) and early fourth (86.00%) waves, until original B.1.617.2 (Delta variant) and related sub-lineages (AY.xx numbered) entered our population, completely replacing the other variants to comprise almost 100% of all sequenced samples by July 2021 [8]. At this point, a new VOC named Omicron has promptly emerged and is putting world health systems in check again [35] due to its higher transmissibility and probably higher resistance to current COVID-19 vaccines. At the time of writing, we are immersed in the sixth wave with 100% positive cases of Omicron in primary health care centers and among patients admitted to our hospitals. The rapid identification of new variants is crucial to health care policies. These examples illustrate the vital role that high quality deep sequencing has played thus far and will continue to play in the future.

In fact, we have learned some lessons from the SARS-CoV-2 pandemic. The first one is that early detection of a novel infectious threat makes it possible to implement control measures when there are few cases, thus increasing the effectivity of actions such as a mass lockdown, which proved to be the most effective tool for stopping the free spread of a highly transmissible respiratory virus. The second lesson is that NGS was essential at the beginning of the pandemic, sequencing the whole genome in a few weeks, from the first case diagnosed on 16 December 2019 to 12 January 2020, when the genetic sequence was publicly shared and uploaded to the NCBI databank on 17 January 2020 (GenBank accession number MN908947.3). SARS-CoV-2 sequencing made it possible to develop highly effective vaccines based on mRNA/DNA platforms, and with many other under development [36] in a record time of six months [37], compared to 34 years for chickenpox, 15 for human papilloma virus, nine for measles, seven for the first polio vaccine, and four for mumps [6,38]. It was also essential to developing qualitative and quantitative molecular diagnostic solutions, identifying variants and relevant mutations for refining new vaccine development, and to studying viral evolution. The third lesson was the power of human data sharing, with millions of sequences already shared in the Global Initiative on Sharing All Influenza Data (GISAID) data-sharing platform [39,40,41]. All these achievements were made possible by the universal application of NGS procedures, especially with Illumina or Nanopore (MinIon) platforms and following the scheme developed by the ARTIC network [42], which have made it possible to sequence millions of SARS-CoV-2 genomes and share them on the GISAID platform. Interestingly, NGS WGS has shown that Omicron sequences are clustered far from the other 5.8M GISAID SARS-CoV-2 uploaded genomes on the evolutionary tree, thus raising the question of which were the predecessors of Omicron [43]. NGS has questioned whether Omicron could have evolved and emerged from a population with little surveillance before jumping to South Africa. It may have been the result of viral evolution in patients with long infection, such as immunosuppressed patients, or as a consequence of a cross-species jump from humans to nonhuman species and then spilling back to humans. This is an open question to which NGS may provide a satisfactory answer. This example highlights the global need to implement molecular solutions using NGS for the rapid detection and characterization of pathogenic viruses. New opportunities are born from the worst crises.

## 3. Zoonosis and NGS

**Figure 2:**
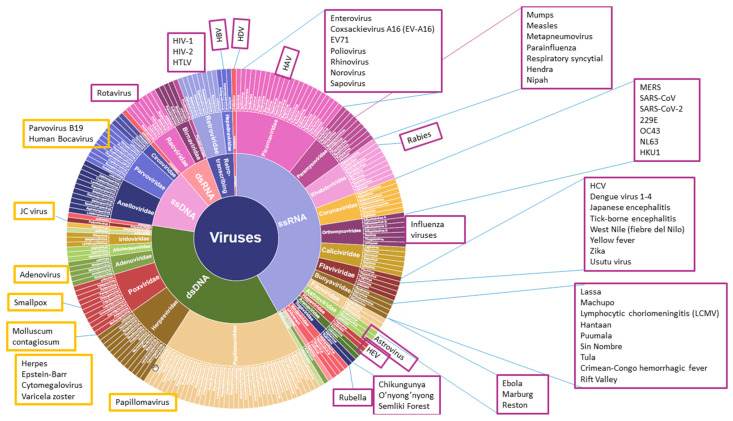
Most human pathogenic viruses in a clock-like classification scheme. Permission kindly provided by Todd N. Wylie; adaptation of Figure 1 of Wylie et al. [52].

Many of humanity’s greatest blights have been caused by viral diseases (Figure 2). The high replication and mutation rates allow the viruses to adapt to different environments, and nearly all of them persist in animal reservoirs. At present, more than 80% of human disease outbreaks are caused by emergent, re-emergent, or new virus infections [44], most of them with a zoonotic origin [45].

Zoonosis is a process through which viruses of wild or domesticated animals and pets can infect humans [46]. Close contact between animals and humans facilitates viral transmissions, although, fortunately, the vast majority of the viruses are not able to infect humans and pass harmlessly through the human gastrointestinal tract or are destroyed by the immune system [17]. While the probability is very low, on rare cases, the virus is able to replicate in a human host. The probability of a viral zoonosis depends on the number of events of cross-species transmissions [17]. Viral zoonosis requires three steps [17,47]: Step 1, the virus begins to replicate in a human subject, leading to viral adaptation and refinement for humans. Step 2, appreciable viral titers in the first human enables infection of a second human, thus initiating selection for viral variants with increased capacity for spreading. Step 3, the virus spreads from human to human. Certainly, an animal virus that never comes into contact with humans has no path for infecting them. In fact, for correct viral replication inside a host cell, viruses must correctly execute ten to hundreds of protein-protein interactions (HIV is thought to interact with as many as 400 proteins in human T cells) [17,48]. Based on the number of animal viruses and those that infect humans, it has been estimated that less than 0.1% of animal viruses progress to step 1 of the process.

The underlying question is whether or not it is possible to predict which viruses have the potential, and which are the genetic changes for the viral adaptation to the human host to accomplish the whole process. Moreover, the challenge is huge, as viruses are not static entities. Indeed, with their large population sizes, short generation times, high mutation rates, and high evolution rates, viruses are ideal evolutionary probes [49,50,51], infecting cells and able to manipulate crucial regulatory nodes of cells to reprogram them into virus-producing factories.

RNA viruses such as smallpox, polio, pandemic influenza, HIV, HBV, HCV, Ebola, SARS-CoV, MERS-CoV, Marburg, Lassa fever, Zika, chikungunya, Nipah virus, hantavirus, avian influenza viruses, and recently SARS-CoV-2 are responsible for an enormous number of human losses and have shaped our biology and our societies. The number of RNA viruses infecting humans may be even higher considering the hit-and-run mechanism, in which the viruses initiate a pathogenic process and then depart without leaving a physical trace of the viral genome [17]. For instance, it has been suggested that some adenoviruses are etiological agents of obesity in animals [53,54,55]. Furthermore, new viruses are constantly being discovered, ranging from gigantic viruses of algae to minimalistic circoviruses. For instance, a hybrid virus was discovered containing genes from papillomaviruses and polyomaviruses isolated from a marsupial called the bandicoot (pig-rat) [56]. Moreover, climate change appears to be favoring the geographical expansion of some viruses (Dengue, Zika, chikungunya, Crimea-Congo, etc.) and their arthropod hosts (ticks, sand flies, and mosquitoes) (Table 1). An example is MERS-CoV in camelids that had an origin in Egyptian tomb bats [57], but seroprevalence studies show a global distribution due to commercial trade of these animals. It has been estimated that ~1001 viruses are known to infect humans [58], and that there are 1,670,000 yet-to-be-discovered viral species in animal reservoirs (mammal and bird hosts) that could infect humans (zoonosis) [59,60]. Thus, more than 99.5% of the potentially pathogenic viruses transmissible from animals to humans are unknown. NGS is the most accurate methodology for identifying new viruses using a random priming approach as described below (see Section 5 and Section 7.1).

In recent decades, the rate of outbreaks of emerging, re-emerging or new infectious human diseases has accelerated due to some factors that mirror the expansion of our global footprint and travel network [61] (Table 1). As Don Ganem put it: “What evolution is operating on is not disease, disease is incidental. It operates on spread” [62]. Viral spread, i.e., the reproduction number (R_0_), primarily depends on factors related to the infectious agent, the host, and the environment: the duration of contagiousness and the likelihood of infection per direct or indirect contact between an infectious agent and a susceptible person, and the contact rate. In this regard, if a poorly spreading virus emerges in a densely populated megacity, it may be spread and refined to infect humans; on the other hand, if a virus excellent at spreading in humans occurs in a small, isolated community in a remote part of the world with a low population density then the virus may falter.

The best approach for predicting a future viral pandemic would be identifying rare animal viruses with thin genetic barriers to replication in human cells, which are the ones that require few mutations to replicate inside human cells. The required constellation of mutations may randomly arise in “natural reservoirs” due to high mutation rates and high population sizes, which increases the probability of cross-species transmission events, and therefore the probability of viral zoonosis. The sequencing of new viruses identified and isolated from animal reservoirs (e.g., bats, birds, rodents) offers limited information for predicting whether one of these new agents is at high risk of spilling over to humans and generating a new outbreak. However, this information may be improved by comparing animal viruses with the viruses that are circulating in the humans. The study of viruses infecting both humans and animals is a novel proposal for identifying the mutations that viruses have acquired during cross-species transmission. Mutations in human viruses circulating in animal reservoirs may jeopardize the effectiveness of vaccines and antivirals and may help predict future epidemics or pandemics.

NGS meets all of these requirements: it can identify individual viral genomes in a complex mixture, thus allowing for the thorough identification of minority variants, which represents a clear advantage over direct (Sanger) sequencing. Sanger sequencing is only able to detect minority variants at frequencies between 10% and 40% and has limited power to sequence complete genomes, with a high associated cost [63]. Direct deep sequencing of clinical material or from natural isolates, either by shotgun or RNA-seq methods, enables WGS of pathogens. This method is being used for diagnosis, molecular epidemiology, metagenomics of viruses, detecting major resistant variants, mapping and predicting epitope changes, and for evolutionary genetics [64,65,66,67].

## 4. NGS for Studying Genomic Viral Variability

RNA viruses, the most prevalent human infectious viruses, which are highly variable agents due to the lack of proof-reading activity of the RNA–dependent RNA-polymerases. Thanks to this, RNA viruses show very high mutation rates, between 10^−3^ and 10^−5^ mutations per nucleotide per genomic replication cycle [68] (i.e., one substitution will be inserted in between 1000 and 100,000 nucleotides copied). This genetic variability can affect some phenotyping features such as tropism, antigenicity, and susceptibility to available antiviral drugs. The mutation rate, also known as the replication error rate, quantifies the number of misincorporations (substitutions) per nucleotide copied and per replication cycle (subst/nt/cycle), which should be measured in viral cell culture experiments [68]. For instance HCV is a single-stranded positive–sense RNA virus 9.6 kb in length and has an estimated mutation rate of 10^−3^ to 10^−4^ subst/nt/cycle [69], which means that every new genome copied will have between 1 and 10 substitutions theoretically inserted at random along its genome. This high mutation rate together with a production and clearance rate of 10^10^ to 10^12^ virions per day in an infected patient [70,71,72] means that a large number of distinct viral variants are produced continuously during infection. The majority of variants produced are cleared by the host’s immune system or are unable to replicate because of a loss of function of encoded proteins [73,74], but a considerable proportion of variants still persist maintaining chronic infection. The frequencies of HCV isolates depend on their replication efficacies and other known and unknown viral and host factors [75,76]. The consequence of the high variation is the continuous production of variants that generates a complex mixture of different but closely related genomes known as quasispecies [77,78]. HCV quasispecies (like any viral quasispecies in general) are composed of a complex mixture of different but closely related genomes whose shape is subject to continuous changes due to both competitive selection [75,79] and cooperation [76] between arising mutations. Differences between those variants may provide them with different phenotypic features, different fitness levels and some of them may have clinical relevance affecting:

- Pathogenesis or virulence;
- Ability to induce peripheral tolerance = escape from natural immune response;
- Escape from vaccination;
- Escape from drug treatment;
- Changes in tissue or host tropism.

HCV is a clear example of the advantage of using deep-sequencing tools to efficiently confront pandemics [4,80,81]. NGS provides solutions for viral subtype classification, to identify mixed infections and identify new subtypes without error [81] and to detect variants resistant to direct-acting antivirals after treatment-failures [4]. This improves treatment effectiveness, reducing antiviral treatment failures and guiding selection of the most accurate re-treatment regimen to eliminate the virus after initial treatment failure, thus significantly reducing the patient’s suffering and health care costs.

CoVs, like most of the other RNA viruses, replicate by means of their own RNA-dependent RNA polymerase (RdRp). However, they have a particularity which is the expression of an accessory nonstructural protein (nsp) 14 (nsp14) which carries RNA proofreading and repair functions because of its 3′–5′ exonuclease (ExoN) activity that can correct mutations introduced by its own RdRp, called nsp12, enhancing replication with high fidelity [82,83,84,85] and thus shows lower mutation rates than other RNA viruses [51,86]. Specifically, for CoVs, mutation rate has been measured as ranging between 2.5 × 10^−6^ for mouse hepatitis viruses and 9.06 × 10^−7^ subst/nt/cycle for SARS-CoV [83,87] much lower than estimated mutation rates from 10^−3^ to 10^−5^ for other RNA viruses. Therefore, it is reasonable to estimate the mutation rate for SARS-CoV-2 in the range between 10^−6^ and 10^−7^. This means that, for SARS-CoV-2, with a length of 30 Kb, it will be necessary to generate 300 new genomes to find one substitution inserted at random along the genome.

Despite this low mutation rate, it is obvious that SARS-CoV-2 is slowly evolving but new variants are continuously arising. In January 2022, 24 clades (19A,B. 20A–J, 21A–D,F–K,L–M) have been reported using Nextstrain classification [9], and 1728 lineages [88] designated using Phylogenetic Assignment of Named Global Outbreak Lineages (PANGOLIN) [85] software. The question is how to explain such a discrepancy between a low mutation rate and the large number of new variants appearing all over the world. In the case of SARS-CoV-2, the low mutation rate is compensated for by a low pathogenicity (most of the infected individuals had an asymptomatic or mild infection), a large population size, in which each infected person carries a total of 10^9^ to 10^11^ virions during the peak of infection with a total mass of 1–100 µg [89], and very high human-to-human transmissibility [90]. At the beginning of the pandemic, with the absence of human pre-existing immunity, the virus rapidly spread through the human population, with very high sequence similarity (99.9%) between isolates recovered all over the world [85], with the increasing amount of infected people and the rate of vaccination, the virus has been subjected to selective pressures and the result is a dramatic increase in the number of variants represented by few mutations. Interestingly, Omicron is increasing in frequency all over the world [91], overcoming the Delta variant that, as previously mentioned, has dominated in recent months and despite the fact that Delta has a high diversification process with more than 200 new AY subvariants [9].

In addition, viruses can exploit several sources of variation other than point mutation (genetic drift), including recombination [92], deletions [34,92,93,94,95,96,97], duplications and insertions. Large genome sequencing using a nanopore approach and short ones using sequencing by synthesis platforms, have made it possible to identify large deletions even from viral isolates on cell cultures and also from natural infections [34,94,95,97,98,99,100]. Sequence deletions during viral RNA replication may be the consequence of the viral replicase jumping on among templates, with deletions occurring in a stepwise manner [101] by slippage of the RNA polymerase when copying an RNA with strong secondary structures, causing the loss of folded RNA fragments. These secondary structures can be affected by substitutions in the RNA [102] and/or by AU-rich stretches acting as hotspot regions for RNA recombination, which may promote the formation of defective RNAs [103]. Some of these deletions resulted in the appearance of a premature in frame stop codon, thus generating defective genomes [104,105]. Among these genomes, those still retaining 5′ and 3′ ends and could be copied and multiplied by the activity of non-defective genomes, thus hijacking their replication machinery complex while co-infecting or superinfecting the same cell. This particular type of defective particles are known as defective interfering particles (DIPs) [106]. DIPs are generated by nearly all viruses [106,107,108] and CoVs are no exception [34,92,93,94,95,96,97]. Since DIPs accumulate at higher rates than full-length viral genomes in the co-infected cells due to their shortened length being more efficiently copied, the amount of standard complete genomes produced is reduced [109], causing a natural interference with the replication, accumulation and transmission of the wild type/complete [107,110,111]. At a high multiplicity of infection, when a cell is infected by more than one genome, defective genomes are encapsulated and secreted outside the cell as a viral particle and DIPs are considered to have clinical potential. NGS is the most accurate tool for identifying such large DIPs that may lately be considered as antiviral tools. However, a proof-of-concept should still be performed.

In addition, viruses can use cellular mechanisms of innate immunity, such as ADAR1 or APOBEC editing activity [112]. Cellular editing activities cause hypermutated full viral genes or genomes [113], usually with deleterious mutations compromising their fitness. In a scenario of low selective pressure, these mutants have a very low probability of being selected and transmitted. However, several viruses have used this genetic mechanism to generate variability that supports increases in infectivity and evolutionary potential [112,113,114,115,116,117,118]. As an example, Hepatitis delta virus uses an ADAR-1 editing mechanism to create the large and short isoforms of the hepatitis delta antigen, both of which are necessary to complete its replication cycle [119]. In the case of SARS-CoV-2, analysis by NGS of its *spike* gene [120] showed that ADAR1 editing was the predominant mechanism generating SARS-CoV-2 genetic variability, although a pattern of nucleotide substitutions suggestive of the ADAR-1 activity was detected only in a small fraction of replicating genomes. However, it seems that the mutations produced by this enzyme were not fixed in the infected human population, suggesting that, by now, nsp12-induced mutations occurring in patients with high viremia and persistent infection would be the main source of new SARS-CoV-2 variants, while those produced by ADAR1 may play an antiviral role [120].

## 5. NGS as a Diagnostic Tool

In recent years, NGS is being introduced into the diagnosis of viral infections, using a metagenomics approach based on random PCR amplification to identify viral genomes present in plasma [121], nasopharyngeal aspirate samples [122], fecal samples [14], and in other body fluids [123,124]. The advantage of using this approach is that each nucleic acid is virtually unbiasedly sequenced; therefore, there is no need to design and synthesize specific primers and probes, thus reducing time consumption, making the identification of new viruses in unknown viral infections possible [125]. Once detected, the new virus or variant identified can be phenotypically studied using, for instance cell culture (when possible) or pseudotyped viral particles [126]. In the case of SARS-CoV-2, this kind of study can test whether the vaccine generated antibodies are able to neutralize the new variant, to determine whether it is necessary to generate neutralizing antibodies for this new VOC detected as a minor variant to modify vaccines, or for antiviral testing [126]. Nevertheless, the random primer approach has a major limitation: correct mapping requires high identity with the reference sequence, minority sequences are underestimated and sometimes not detected [127]. This limitation does not allow for the surveillance of variants potentially present in very low proportions in the quasispecies of some infected individuals. These minority variants may subsequently become dominant and represent a public risk. More detailed information on WGS random priming strategy and their advantages and disadvantages is provided later (see Section 7.1).

In contrast, a deep NGS approach using specific primers can detect these minor variants in the quasispecies or detect mixed infections [81]. This approach may be used to demonstrate viral transmission from person to person in cases where transmission has occurred after bottlenecking, in which a subpopulation of the source of the virus has started the new infection in the donor [128,129]. In our center, NGS using a specific primers approach has been transferred to healthcare diagnostic practice for high-resolution HCV subtyping as an error-free method able to classify all viral isolates, identify new subtypes, and report whether the patient is infected by more than one subtype at the same time (mixed infection) [80,81]. This test is based on a robust methodology of RT-PCR-Nested amplification that can amplify all HCV subtypes. The RNA is RT-PCR-Heminested amplified using specific and patented primers of the NS5B region and deep-sequenced using an NGS platform. NS5B raw sequences are lately subjected to a filtering process and phylogenetic classification using reference HCV genomes sequences [130,131,132,133]. More recently, a test for resistance-associated substitutions (RAS) has been reported. Assessment of minority variants harboring RAS makes it possible to predict their selection with specific direct acting antivirals against HCV [63,134]. This allows for the creation of a final report for RAS that includes recommendations for re-treatment of patients refractory to the antiviral treatments [135]. More detailed information on the specific primers strategy to study viral quasispecies and its advantages and disadvantages is provided below (see Section 7.3).

## 6. NGS for Studying the Origin of a Virus and Fighting against Fake Theories

The origin of SARS-CoV-2 is still in the spotlight but there is no doubt about its zoonotic origin [136,137,138]. NGS was useful in obtaining the whole genome of original Wuhan-hu-1 SARS-CoV-2 genome and it was later used to upload millions of sequences from isolates all over the world [40]. Comparing Wuhan-hu-1 and the closest CoV isolate from any animal (bat CoV RaTG13) shows that 3.25% of their genome is different (more than 900 nts out of 29,813), while only 0.36% (107 nt changes out of 29729) of the genome is different when comparing Wuhan-hu-1 and an Omicron isolate from the city of Barcelona (calculations based on authors own data). Two close examples of a viral zoonotic origin are the two preceding CoVs epidemics, SARS-CoV and MERS-CoV [94,97,98,100,101,139]. SARS-CoV genomes of viruses isolated from marketplace masked palm civets, raccoons, dogs, and ferret badgers are almost identical to those isolated from humans [102,103,104], showing that these animals were the intermediate hosts between the original CoVs infecting bats (*Rhinolophus affinis*, *Rhinolophus sinicus*) and humans. Human isolated MERS-CoV have greater than 99% identity with those isolated from dromedary camels [16], which were the intermediate host between the original CoVs infecting the Egyptian tomb bat and humans. On the other hand, the divergence of SARS-CoV-2 in relation to other CoVs is much larger. This CoV shows the insertion of four polybasic amino acids (PRRA) at the furin cleavage site, and five amino acids (F484, Q493, S494, N501 and Y505) changed in the receptor binding domain in relation to the closest RaTG13 [136], and other substitutions which have not been reported before in bats and other CoVs [136,140,141]. All those insertions and amino acid changes shown along the spike gene that facilitated viral spread through the human population were not obvious to any expert. Besides, no previously cultured CoVs had shown anything similar to those genome modifications [142], thus constituting another strong argument that this virus could not have been created by any researcher in a lab. The most probable explanation for the accumulation of characteristic amino acid insertions and changes observed in the SARS-CoV-2 genome is that they are a result of multiple jump events from animal to human (zoonosis) and from human to animal (zooanthroponosis) [143], in which the virus may have evolved in both species with the subsequent spread among the human population in the absence of pre-existing human immunity against SARS-CoV-2 [34]. In fact, it is highly improbable that a cross-species jump resulting in a human pandemic could happen as a single event, as explained in Section 3.

NGS was essential to demonstrating that outbreaks of SARS-CoV-2 on mink farms in Denmark and The Netherlands originated in humans. In these outbreaks, the virus was initially introduced by humans, evolved in minks and spilled over to humans [144,145,146]. Although the virus has been able to jump from human to zoo lions and domestic animals such as dogs and cats, and while there is evidence for efficient transmission, the likelihood of pets or felines as the origin of the pandemic has been ruled out and it has not been considered as a major concern [147,148,149]. In addition, this is not restricted to SARS-CoV-2 because a case of a novel canine CoV transmission from domestic dogs to humans has been reported in Malaysia [150]. Therefore, we must be aware and avoid mass infection in these animals so as not to facilitate viral adaptation with unpredictable results [17,143]. To study the origin of SARS-CoV-2, it would be interesting to use NGS to compare sequences isolated from wild animal farms used for fur and food production, such as those dedicated to farming mink, foxes, and raccoons, which have been on the rise in China since the 1990s (nearly 60 million animals were slaughtered in 2018) [151]. Indeed, the SARS-CoV-2 pandemic has reinvigorated the One Health approach [61], as defined by Centers for Disease Control (CDC): “One Health recognizes that the health of people is connected to the health of animals and the environment” [152]. In the case of infectious diseases, this means that we should develop multisector and transdisciplinary strategies to integrate NGS sequencing studies of infectious agents isolated from humans, animals, plants and environments with biomedical sciences, bioinformatics, veterinarian and medical professionals of food and environmental sciences.

## 7. NGS Methodologies for Emerging, Re-Emerging, and New Viruses

**Figure 3:**
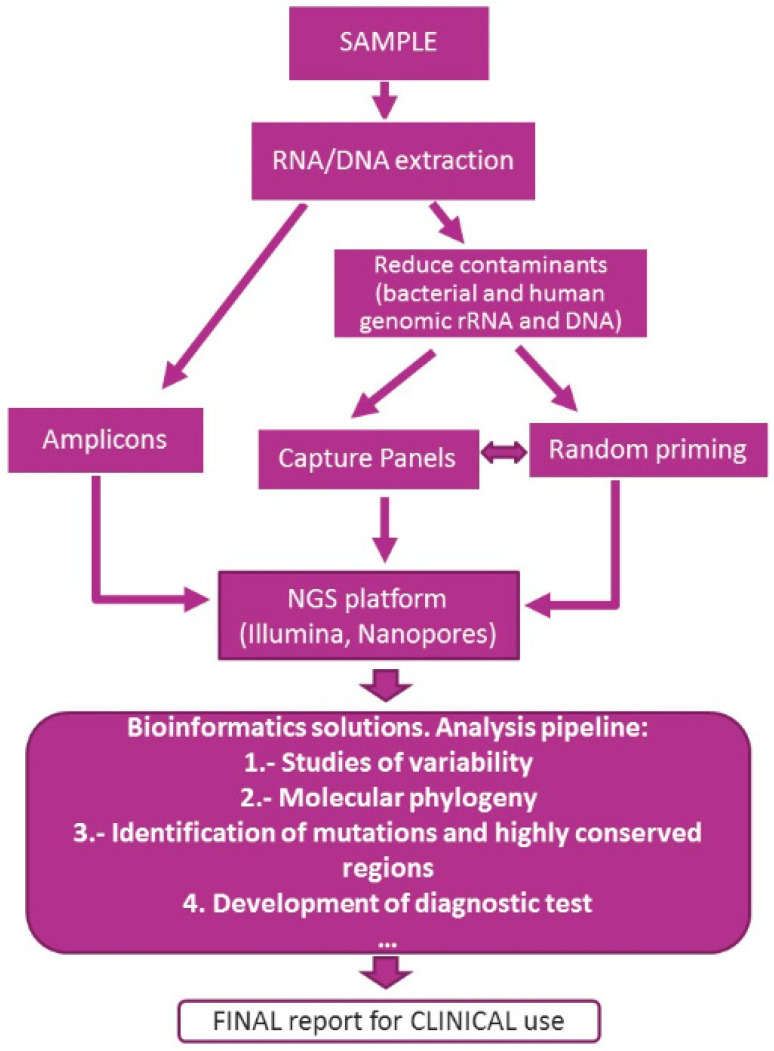
General schematic methodologies for virus genome sequencing.

General methodological strategies for NGS viral sequencing are summarized in Figure 3.

### 7.1. NGS Using Random Primers (Metagenomics Sequencing)

Random primers are short single-stranded DNA oligonucleotides only six to nine nucleotides long (usually hexamers), and they consist of every possible combination of bases, which means that a mixture contains 4096 different hexamers. As all possible hexamers are present, these primers can bind to any RNA or DNA sequence target in a mixture of genomes. Then, any retrotranscription will copy any RNA genome to DNA, and in a PCR, any DNA fragment will be amplified. The PCR product might be successfully sequenced after composing a library to be loaded and run in an NGS platform.

This technical solution has important advantages:

- It does not require prior knowledge of the genome for primer or probe design, which simplifies the process overall;
- It allows for the identification of all pathogens in any kind of sample (plasma, serum, feces, cerebrospinal fluid, sewage, etc.) as well as the major genomes of the viral population in epidemiological studies, or outbreak investigations.

However, WGS with random priming has an important disadvantage: it amplifies any DNA or RNA genome at random, and, as a result, minority genomes are underrepresented or even lost (lower sensitivity), since nucleic acids from the host and commensal microorganisms in clinical specimens will also be amplified. The huge number of unwanted amplifications makes the cost of obtaining sufficient data high, which increases the computational challenge. The proportion of reads that match the target virus genomes is commonly very low: 0.008% for Epstein—Barr virus (EBV) in the blood of a healthy adult, 0.0003% for Lassa virus in clinical samples, and 0.3% for Zika in a sample enriched for viral particles through filtration and centrifugation [127]. Therefore, random priming requires a large quantity of starting genomic material (e.g., high viral load).

A possible solution to this limitation is to attempt to concentrate the infectious agent prior to the sequencing methodology. This can be done using antibodies, filtration, ultracentrifugation, and depletion of free nucleic acids that mostly come from the host. Viral RNA could be further enriched by the depletion of host material such as ribosomal RNA (ribosomal depleting protocols), bacterial and host DNA (DNAse treatment and filtration), and random primers could then be used to increase the amplified genomic yield [153,154].

### 7.2. Sequence Capture, Fragment Recovery and NGS Sequencing (Target Enrichment Sequencing)

A method for pathogen enrichment consists of designing small RNA or DNA overlapping probes complementary to the pathogen reference sequences. They are typically attached to a solid phase (for example streptavidin-labelled magnetic beads) and used to capture or “pull down” complementary DNA sequences from total nucleic acids in a sample. DNA damage is repaired (nicks, thymidine dimers, oxidized guanines, and pyrimidines, etc.) and end repaired. Adaptors are then ligated and sequences enriched using few PCR cycles [66].

Advantages:

- The sequence capture strategy allows for enrichment of sequences from specific microorganisms in a complex mixture;
- Whole genomes may be sequenced at a low cost, as non-pathogenic genomes are significantly reduced, thus reducing the contaminating nucleic acids from different origins. Thanks to this, few PCR cycles are necessary for amplification, limiting the introduction of artefact mutations, and minor variants are preserved (i.e., higher sensitivity), reflecting in vivo variation.

Disadvantages:

- The design of oligonucleotide capture probes requires knowledge of the microorganism genome. Thus, it does not allow for the sequencing of novel pathogens;
- It requires high technical expertise for sample pre-treatment;
- Although it is highly sensitive, the recovered sequencing coverage is low.

### 7.3. Use of Amplicons (PCR Amplicon Sequencing)

The use of overlapping amplicons consists of designing specific primers to amplify a partial or complete genome by PCR which is the most widely used sequencing approach in our laboratories [4,80,81,155].

Advantages:

- It is a highly sensitive and specific method, widely used, with trusted and well-established methods;
- It is able to specifically amplify the infectious agents, significantly decreasing the non-pathogen genomic background, and increasing cost-effectiveness per sample.
- It achieves good coverage even at a low pathogen load;
- It is the most sensitive method for genome sequencing from a complex mixture, thus, allowing for the detection of variants at very low frequencies.

Disadvantages:

- It is highly dependent on the primer’s quality, specificity, and mismatch, particularly in poorly characterized pathogens, in pathogens with recognized high genetic diversity or those with novel variants;
- The higher the number of cycles, the higher the number of artefact mutations;
- It requires knowledge of the microorganism genome to design specific primers.

### 7.4. Next-Next Generation Sequencing (NNGS) or Third Generation Long-Read Sequencing (TGS)

The next-generation sequencing technologies have been classified according to the read length, when they are short as “second generation” or “NGS” technologies, and when they are long as “third generation” or “NNGS”, technologies (Table 2).

NGS short-read sequencing are currently represented by Illumina (Illumina Inc., USA) and Ion Torrent (ThermoFisher Scientific, USA), while 454 sequencing technology (Roche, USA) was discontinued in December 2016. NGS has been extensively used for viral research and also for clinical virology applications [155,156,157]. The main drawbacks of NGS technologies are the short length of the reads (75–600 nts/read) that restricts the possibility to identify structural variants, large deletions or insertions, settle repetitive elements and/or sequences with extreme guanine-cytosine (GC) content, and to identify whether different mutations are associated in the same genome. The combination of mutations (constellation of mutations) in the same genome is of particular interest in virology, as some substitutions affecting functional elements can be compensated by mutations in other parts of the genome that allow the viral RNA or the protein, for instance, to reduce the susceptibility to an antiviral or to change antigenic properties recognized by the acquired immune response, but remain functional.

Third-generation sequencing (TGS), also known as long-read sequencing [153,159,160], has the great advantage of reporting large and extremely large reads, with hundreds of kilobases in one single read to extremely large reads, to even close to 1 million base pair reads. TGS is achieved through single-molecule real-time (SMRT) technology (Pacific Biosciences, USA) [154,161] and the nanopores technology provided by Oxford Nanopore Technology (Oxford, UK), Quantapore Inc. (Sand Francisco, CA, USA) and Stratos (Roche, USA) which basically consist in passing a DNA molecule or a DNA surrogate though a nanopore and then measuring electrical field changes. One of the major achievements of TGS, after 20 years of sequencing, has been to finally sequence the complete human genome from end to end [162], another is the direct sequencing of the RNA genome of influenza A virus [163] thus allowing one to report information on the RNA modifications, and also direct detection of DNA methylation due to their distinctive signal from the other four nucleotide bases [164].

Drawbacks of TGS (NNGS) are the systematic sequencing errors due to problems on the consistency of the electric signals, difficulties in obtaining multiple overlapping reads, the large amount of genetic material required for direct sequencing, and the low throughput. However, continuous improvements augur a brilliant future for this technology for research and diagnosis in the field of genetic and infectious disease studies.

## 8. Summary

A common trait among viruses, especially RNA viruses, is their elevated ability to generate genetic variability, thus leading to a scenario in which we cannot talk of a virus as a sequence but as a population of genomes (i.e., a quasispecies). Any source of variation provides a minor viral reservoir of genomes that may be selected in the face of environmental selective pressure. Genetic variability (single point mutations, insertions, deletions, recombination, and reassortment events), as well as host innate immune system genetic edition mechanisms (ADAR, APOBEC) can affect some phenotypic features such as tropism, antigenicity, virulence, and susceptibility to available vaccines and/or antiviral drugs. The best tool for obtaining all these data and detecting, identifying, and characterizing a viral agent and even detecting minor variants, is undoubtedly the NGS technology, based on either partial or whole genome sequencing of any viral agent. In the present SARS-CoV-2 pandemic, NGS has been crucial to developing molecular assays for its detection (PCR), to support the development of effective vaccines (mRNA- and DNA-based vaccines), detecting, monitoring and characterizing circulating variants, identifying essential viral proteins for designing antivirals (direct-acting antiviral drugs), screening for mutations impacting on transmissibility and/or on pathogenicity, understanding the viral life cycle (identifying weaknesses), studying the origin of pandemics and screening for potential reservoirs, performing nosocomial and community outbreak as well as spill-over event investigations.

## Figures and Tables

**Table 1:** Summary of factors related to a higher probability of zoonosis.

Factor	Examples
Degradation of natural and wild spaces	Deforestation.
	Dam construction. Changes in wildlife migration patterns. Floods due to changes in river boundaries. Global warning, climate change causing the expansion of viral vectors. Agriculture.
Bringing humans closer to wild animals, putative viral reservoirs	Wild animal parks.
	Hunting.
	Tourism/Travel to exotic areas.
Globalization effects	Human mobility losing the quarantine effect.
	Commercial trade.
	Long distance transport of birds and livestock.
	Urbanization (concentrating millions of people in small places).
Massive exploitation of animals	Pigs, chicken, birds, livestock, including wild animal farms for fur and food production.
Health and social activities	Transfusions, organ transplants.
	Social changes related to sex and drug abuse.
	Large concentrations in closed halls, stadiums, pavilions.

**Table 2:** Comparison between second and third generation sequencing methodologies. Advantages and drawbacks [158]. * Taking Ion Torrent Personal Genome Machine and Illumina MiSeq platforms as examples. ** Taking Pacific Biosciencies RSII and Oxford Nanopore MinION platforms as examples. *** Viral haplotypes = unique sequences in the quasispecies, corresponding to a quasispecies variant.

	Second Generation *	Third Generation **
**Read length**	400–≈500 (2 × 300) bp	60 K–2 M bp
**Error rates**	≈1%–2.4%	≈10%–15%
**Advantages**	**High throughputs with relatively low error rates** makes them suitable for: Quantitative detection of variants (even minor ones). Study quasispecies diversity (complexity of variant mixture).	**Much longer reads** enable more accurate haplotype *** reconstruction, which allows: Long-range linkage between different genomic regions. Improved genotype classification, especially to distinguish between mixed/recombinant genotype in HBV infection.
**Disadvantages**	**Short reads** are poorly suited for: Assembly and characterization of complex/highly repetitive genomic regions. Reconstruction of complete “real” viral haplotypes ***.	**Higher error rates** difficult characterization of “real” nucleotide substitutions, insertions and deletions.

## Data Availability

Not applicable.
